# Randomized Controlled Study Comparing Disposable Negative-Pressure Wound Therapy with Standard Care in Bilateral Breast Reduction Mammoplasty Evaluating Surgical Site Complications and Scar Quality

**DOI:** 10.1007/s00266-018-1095-0

**Published:** 2018-02-13

**Authors:** V. Tanaydin, J. Beugels, A. Andriessen, J. H. Sawor, R. R. W. J. van der Hulst

**Affiliations:** 1 Maastricht University Medical Center, Maastricht, The Netherlands; 2 Andriessen Consultants, Malden, The Netherlands; 3 UMC St Radboud Nijmegen, Nijmegen, The Netherlands; 4 VieCuri Medical Center, Venlo, The Netherlands

**Keywords:** Negative-pressure therapy, Post-mammoplasty, PICO, Scar, POSAS, Cutometer

## Abstract

**Background:**

Negative pressure wound therapy (NPWT) for postsurgical incision treatment has demonstrated benefits. A prospective randomized study was developed including 32 patients who underwent bilateral breast reduction mammoplasty. Patients served as their own control and received NPWT to one breast and fixation strips to the other breast.

**Methods:**

The primary outcome was the number of wound healing complications within 21 days when comparing NPWT treatment with fixation strips. The secondary outcome was aesthetic appearance and quality of scarring using questionnaires [visual analogue scale (VAS) and Patient and Observer Scar Assessment Scale (POSAS)] scored at day 42-, 90-, 180- and 365-day follow-up using additional scar measurement modalities, such as viscoelasticity.

**Results:**

For the 32 included patients, the number of wound complications was significantly lower (*p* < 0.004) for the NPWT treated sites compared to fixation strips. POSAS and VAS scores at 42 and 90 days revealed a significantly better quality of scarring in the NPWT treatment breasts than in fixation strips. At 180-day follow-up, there was a significant improvement in VAS scores, as well as a comparable improvement in POSAS scores. No consistent significant improvement in scar quality was demonstrated with the assays that were used.

**Conclusions:**

Our study showed less complications and a significant improvement in quality of scarring in favor of the NPWT-treated sites. The results indicate NPWT to be an attractive option for these patients.

**Level of Evidence II:**

This journal requires that authors assign a level of evidence to each article. For a full description of these Evidence-Based Medicine ratings, please refer to the Table of Contents or the online Instructions to Authors www.springer.com/00266.

## Introduction

Breast reduction is a common cosmetic surgical procedure to reduce the size of the breast and to overcome discomfort caused by oversized, ill-shaped and hanging breasts [1]. Although most complications can be overcome with proper selection of the procedure and with gentle tissue handling, reported percentages of complications are as high as 53% [2–4]. The most common complication is delayed wound healing, and other complications include hematoma, fat necrosis, nipple necrosis, cellulitis and fungal dermatitis [4–6]. Hypertrophy is common after inferior pedicle breast reduction in the inframammary scar, and 15% of all scars are reported to be thick, itchy or uncomfortable [2]. The assumption is that quality of scarring may be improved when wound healing is effective, without complications. Negative pressure wound therapy (NPWT) refers to the controlled application of sub-atmospheric pressure to promote wound healing and has been applied in a wide array of acute and chronic wounds [7–9]. Over the years, clinical success of NPWT encouraged specialists to apply the technique to closed surgical incisions. It serves as a preventative measure after high-risk procedures, mainly in trauma and cardiothoracic surgery, or in patients with multiple comorbidities and/or risk factors such as obesity and use of steroids [10, 11]. Stannard et al. [12, 13] published two studies on different high-risk skeletal traumas in which they concluded that there were significantly less infections and cases of dehiscence in the NPWT group compared to the sites treated with standard care. A recent development in the field of NPWT is a portable disposable system. An early study evaluating the performance and clinical benefits of the system showed promising results [14].

A study was developed including 32 patients undergoing bilateral reduction mammoplasty, to evaluate the effectiveness of post-surgery incision treatment comparing a portable disposable NPWT system with standard care, using fixation strips. Patients were followed up to 365 days post-surgery.

## Methods

### Study Population

The prospective randomized, controlled, comparative study included 32 patients > 18 years of age, who underwent bilateral superomedial pedicle Wise-pattern breast reduction mammoplasty and had postsurgical incisions of similar length on each breast. Patients received treatment at VieCuri Medical Centre in the Netherlands. Exclusion criteria included pregnancy or lactation, using steroids, or other immune modulators known to affect wound healing; history of radiation of the breast; tattoos in the area of the incision; skin conditions such as cutis laxa that would result in poor healing or widen scars history of radiation of the breast, patients with a known significant history of hypertrophic scarring or keloids, and postsurgical incisions still actively bleeding, exposure of blood vessels, organs, bone or tendon at the base of the reference wound; and incisions > 12 inches (30 cm) maximum linear dimension.

#### Study Design

This was a level II therapeutic study. The patients served as their own control, with both breasts included in the study. For this long-term (365 days) follow-up study, the sample size available for analysis (*n* = 32) represents the number of patients enrolled into the larger multicentre RCT at VieCuri Medical Centre in the Netherlands. No a priori sample size calculation was performed. As the long-term follow-up was a single-center study, this number of patients allowed us to keep recruitment time and study duration manageable. Post hoc sample size calculations using nQuery 4.0 confirmed that the present study had sufficient (greater than 80%) statistical power to detect a difference between NPWT and standard care, based on the data observed, in terms of the scar quality and aesthetic appearance outcomes (VAS and POSAS) in the shorter-term, 42 and 90 days, follow-up. Further post hoc sample size calculations showed that the same outcomes at the longer-term follow-up, 180 and 365 days, would require a larger study ranging between *n* = 50 and many hundreds of patients, depending on outcome and time point, to be sufficiently powered.

Each patient received postsurgical treatment with both NPWT and standard care. Randomization was used for allocation of NPWT and fixation strip to the right or left breast incision site per patient, using sealed envelopes. Treatment site information was accessed digitally (www.sealedenvelope.com) upon the start of the treatment postsurgically. As NPWT and fixation strips are optically different, blinding of the physician and patients was not feasible; however, data analysis was performed blinded. All included patients (*N* = 32) had follow-up visits and assessments at screening (pre-surgery), day 0 (baseline, post-surgery), day 7, 21, 42, 90, 180 and 365 days post-surgery (Fig. 2).

#### Study Interventions

**Fig. 1:**
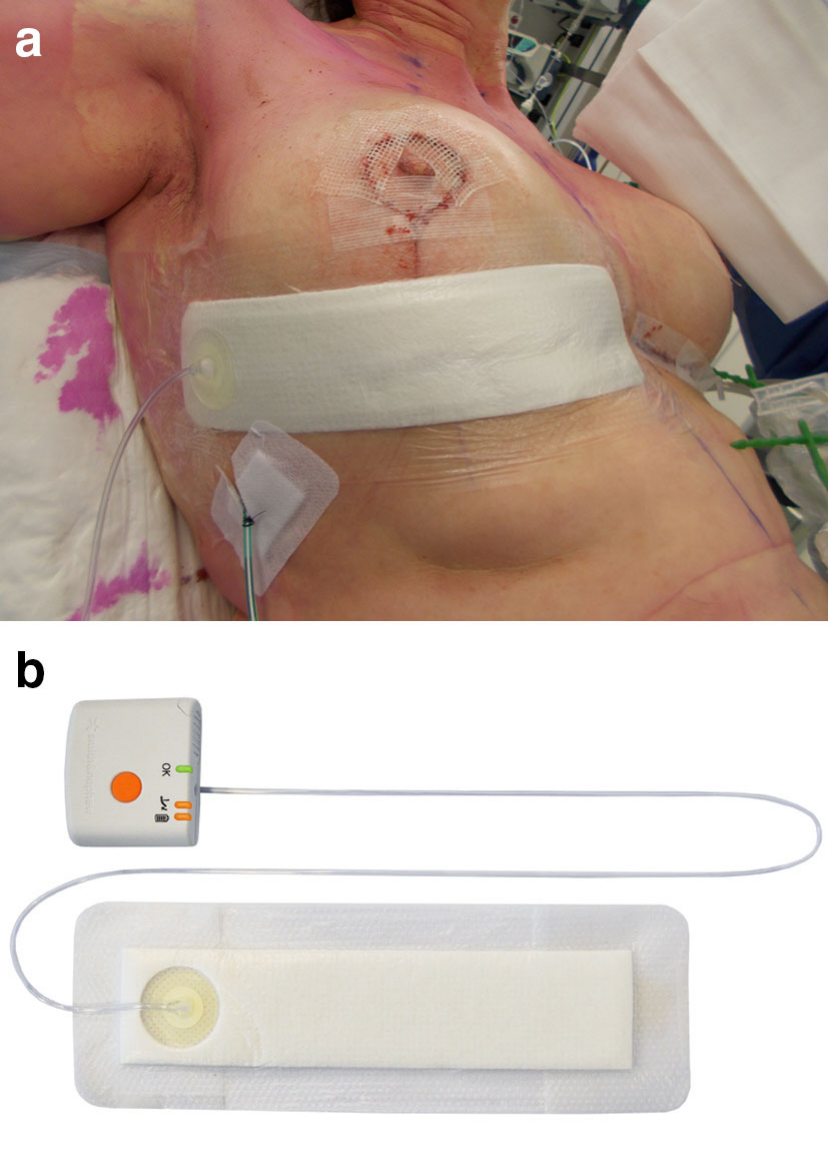
**a** The PICO dressing in place on lower horizontal incision. **b** PICO pump. Reprinted with permission of Smith and Nephew

The study product is a single-use NPWT system without an exudate canister, allowing the portable pump to be small and light weight (PICO, Smith and Nephew Medical Ltd, UK). It is indicated for use on both open and closed wounds with low to moderate levels of exudate and comes with two adhesive dressings and ten fixation strips (Fig. 1a). The pump weighs 70 grams, uses two AA batteries and is capable of delivering 80 mmHg negative pressure to the wound surface. A single start/pause button (with automatic restart after 1 h) and alarm LEDs for leak and low battery enable easy use (Fig. 1b). The system, without canister, is able to manage low to moderate levels of exudate generated by the wound, mainly by evaporation through the specially designed multilayer dressing. The system is programmed to provide therapy for up to 7 days. Various size dressings are available.

As standard treatment, fixation strips (STERI-Strip, 3M, St. Paul, MN, USA) were used. These sterile skin closure strips have a porous, non-woven backing coated with a pressure-sensitive, hypoallergenic adhesive and is reinforced with polyester filaments for added strength.

#### Study Outcomes

The primary outcome of the study was the number of surgical site complications within 21 days post-surgery when comparing NPWT versus standard care using fixation strips. The secondary outcome was aesthetic appearance and quality of scarring, assessed at day 42, 90, 180 and 365 days.

#### Assessment Tools

##### Questionnaires and Scales

Clinicians assessed the surgical site condition for wound healing status at 21 days, scoring on a clinical scale. The cosmetic result and scar quality were assessed using two different types of questionnaires.

##### The Patient Scale and Observer Scale

The Patient Scale and Observer Scale (POSAS) is a comprehensive scale designed for the evaluation of scar quality by professionals (observer scale) and by patients (patient scale). Both scales contain six items that were scored numerically and made up a “total score.” The POSAS has category boxes to score nominal parameters (e.g., color of the scar). Each item of both scales had a 10-point score, with 10 indicating the worst imaginable scar or sensation and 1 corresponding to normal scar or skin (normal pigmentation, no itching, etc.). The total score of both scales was calculated by summing up the scores of each of the six items and ranges from 6 to 60. The patient and observer also scored their “overall opinion” [15, 16].

##### Photograph-Based Scale

The multi-category visual analogue scale (VAS) is a photograph-based scale derived from evaluating standardized digital photographs in four categories (pigmentation, vascularity, acceptability and patient comfort) plus contour. It sums up the individual scores to get a single overall score ranging from “excellent” to “poor.” It demonstrated high observer reliability and internal consistency when compared to expert panel evaluation, but has only moderate reliability when used among lay panels [17–19].

Additionally, scar assessment was performed by placing a vertical mark on a 10-cm VAS line to represent the scar. For the purposes of study data collection, the line was divided into 10 boxes, to represent a 10-point scale, to enable accurate recording and interpretation of the data. The sum of these scores then forms the overall scar score.

#### Quantitative Scar Measurements

**Fig. 2:**
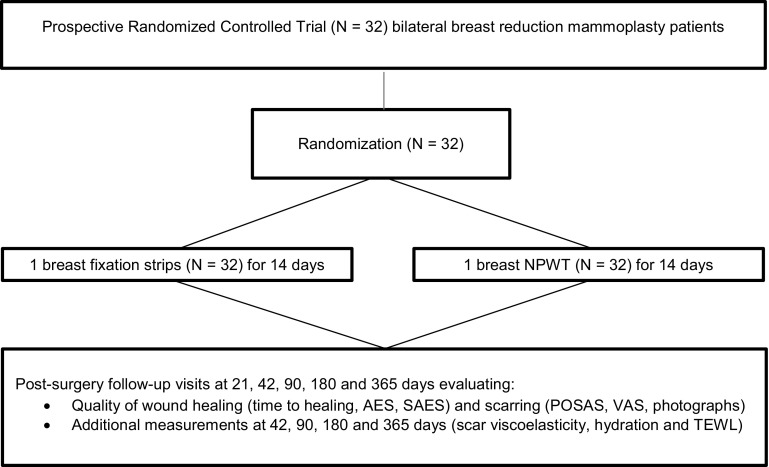
Schematic profile of study disposition. NPWT, negative pressure wound therapy; AES, adverse events; SAES, serious adverse events

To obtain quantitative information on scar quality, the Cutometer^®^ MPA 580 system (Courage + Khazaka electronic GmbH, Cologne, Germany) was used. The system has a wide variety of probes measuring different skin characteristics. For the study, three probes were used to assess scar viscoelasticity, water content level of the upper skin layers, and trans-epidermal water loss (TEWL). The measurements were recorded in a data file (MS Excel) [20–22]. POSAS, VAS scores and quantitative scar measurements took place during follow-up visits on day 42, 90, 180 and 365 (end of study) (Fig. 2).

#### Data Collection and Analysis

The POSAS and VAS scores were summarized at the 42-, 90-, 180- and 365-day follow-up assessment. For each treatment, the presence of diabetes, whether the patient smokes, and body mass index were taken into account. Statistical evaluation was performed using IBM SPSS. A paired *T* test was used to calculate the difference between treatments for POSAS and VAS scores. If the differences between treatments were not normally distributed, the Wilcoxon signed-rank test was used. Tests were carried out at the 5% significance level and a confidence interval of 95%.

The measurements for viscoelasticity, water content and TEWL were transferred from the device’s software to a database and analyzed using IBM SPSS. The standard locations for measurements with the device were: (a) normal skin between the breasts just below the sternum as reference (b) on the horizontal scar 5 cm laterally of the vertical scar in each breast.

### Ethical Issues

All procedures performed were in accordance with the ethical standards of the institutional and/or national research committee and with the Declaration of Helsinki and its later amendments or comparable ethical standards. Institutional review board approval was obtained. The study registration ID number is NL40698.068.12/METC 12-3-026. The start date of the study was June 1, 2012, and the completion date was April 9, 2014.

Patients were assessed according to the eligibility criteria and were provided with study information and adequate time to read and understand the information. Inclusion in the study followed after the patients had received answers to their questions and gave written consent.

## Results

The included patients had a mean age of 40.9 years, ranging from 18 to 61 years (Table 1). All patients were Caucasian, had American Society of Anesthesiologists (ASA) Physical Status classification scores of 1 or 2 and did not have diabetes mellitus. Of the 32 patients, 28 (87.5%) did not have a medical history prior to the surgical procedure. Only two patients (6.25%) smoked approximately 5 cigarettes a day. Four patients (12.5%) used medication; however, none of the medications are known to influence wound healing.

**Table 1:** Baseline characteristics for *N* = 32

	Mean (range)
Age	40.9 (18–61)
BMI	26.5 (19.5–31.2)
Cup size	E–F (E–I)
Clavicle-areola distance	28.2 cm (23–35)
Resection weight	447.0 g (113–1260)
Duration procedure	97.8 min (49–157)
Length of incision	19.7 cm (13–24)

### Surgical Site Complications

Follow-up assessments were carried out to evaluate the difference in incision healing complications between the NPWT and fixation strips treated sites, up to 21 days post-surgery. Wound healing complications were defined as delayed healing (surgical incision not 100% closed at day 7 post-surgery), or occurrence of dehiscence or infection within 21 days post-surgery. Superficial wound dehiscence occurred in 10 (31.3%) patients. There was significantly less dehiscence (*p* < 0.001) for the breasts treated with NPWT compared to the sites treated with fixation strips. Of the patients who presented with wound dehiscence, 5 (15.6%) had this complication in both breasts.

Of those five patients who had bilateral wound dehiscence, in 2 (40%) patients the NPWT-treated site healed faster than the site treated with fixation strips. Unilateral wound dehiscence occurred in 5 patients, who all had post-surgery treatment with fixation strips on this incision site. The total number of wound complications was significantly lower (*p* < 0.004) for the NPWT-treated breasts.

#### Scar Quality and Aesthetic Appearance

**Fig. 3:**
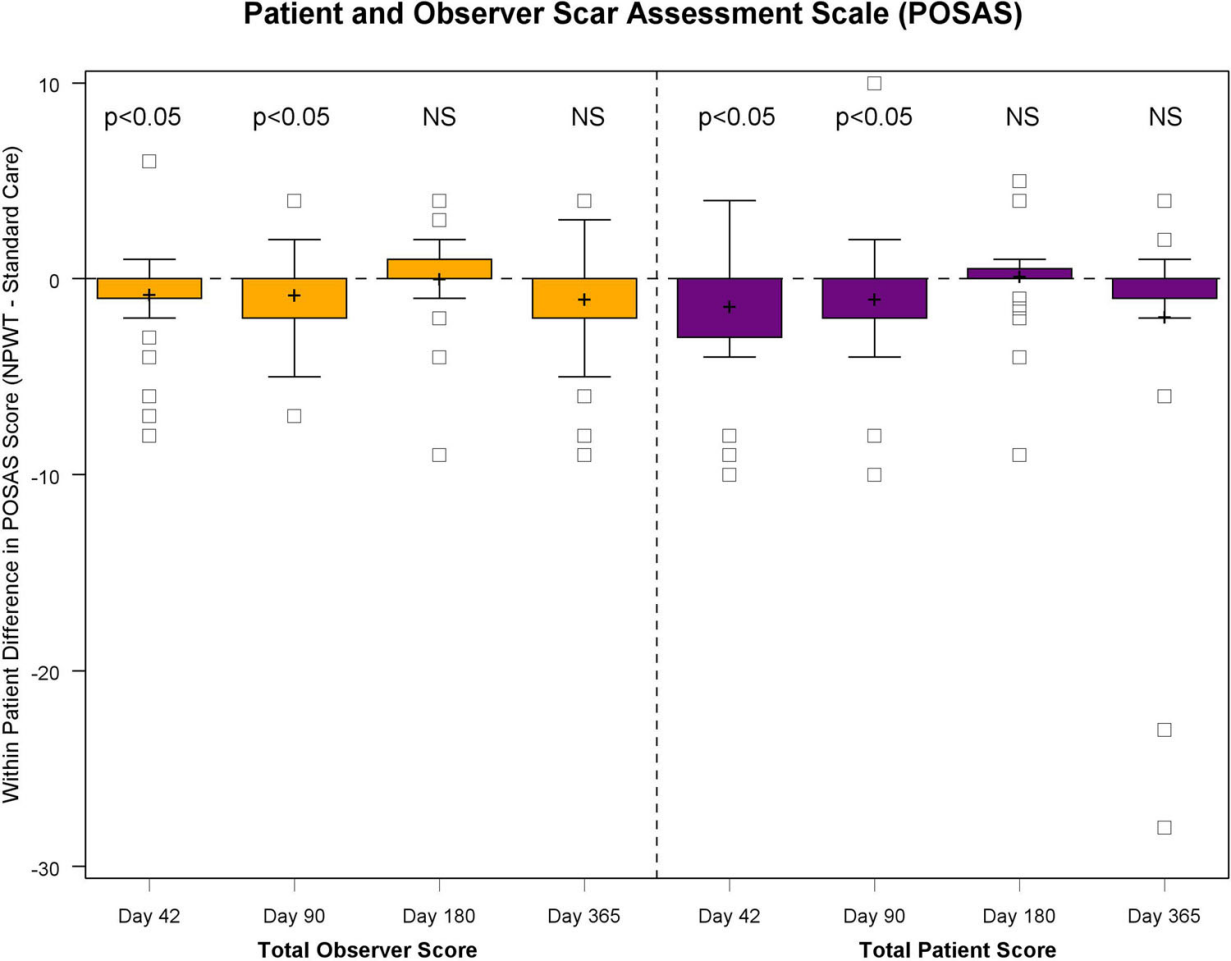
*N* = 32 scar quality POSAS scores at 42, 90, 180 and 365 days. POSAS, patient and observer scar assessment scale

**Fig. 4:**
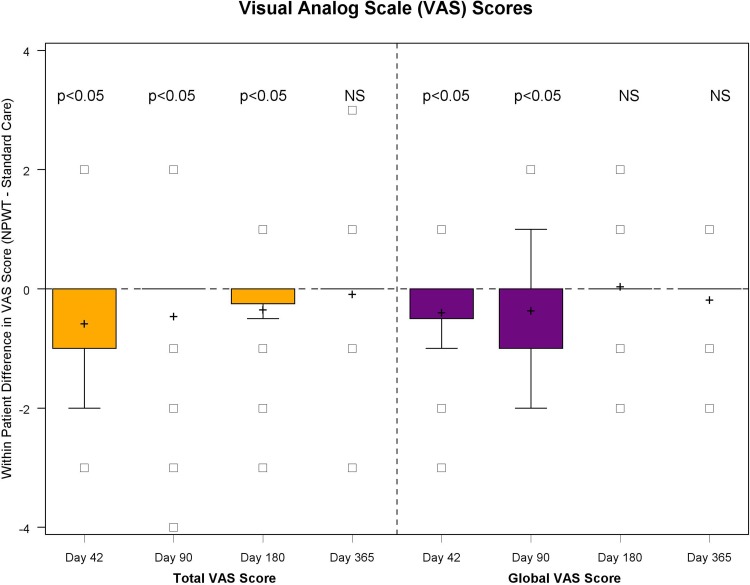
*N* = 32 VAS scored at 42, 90, 180 and 365 days. VAS, visual analog scale

**Fig. 5:**
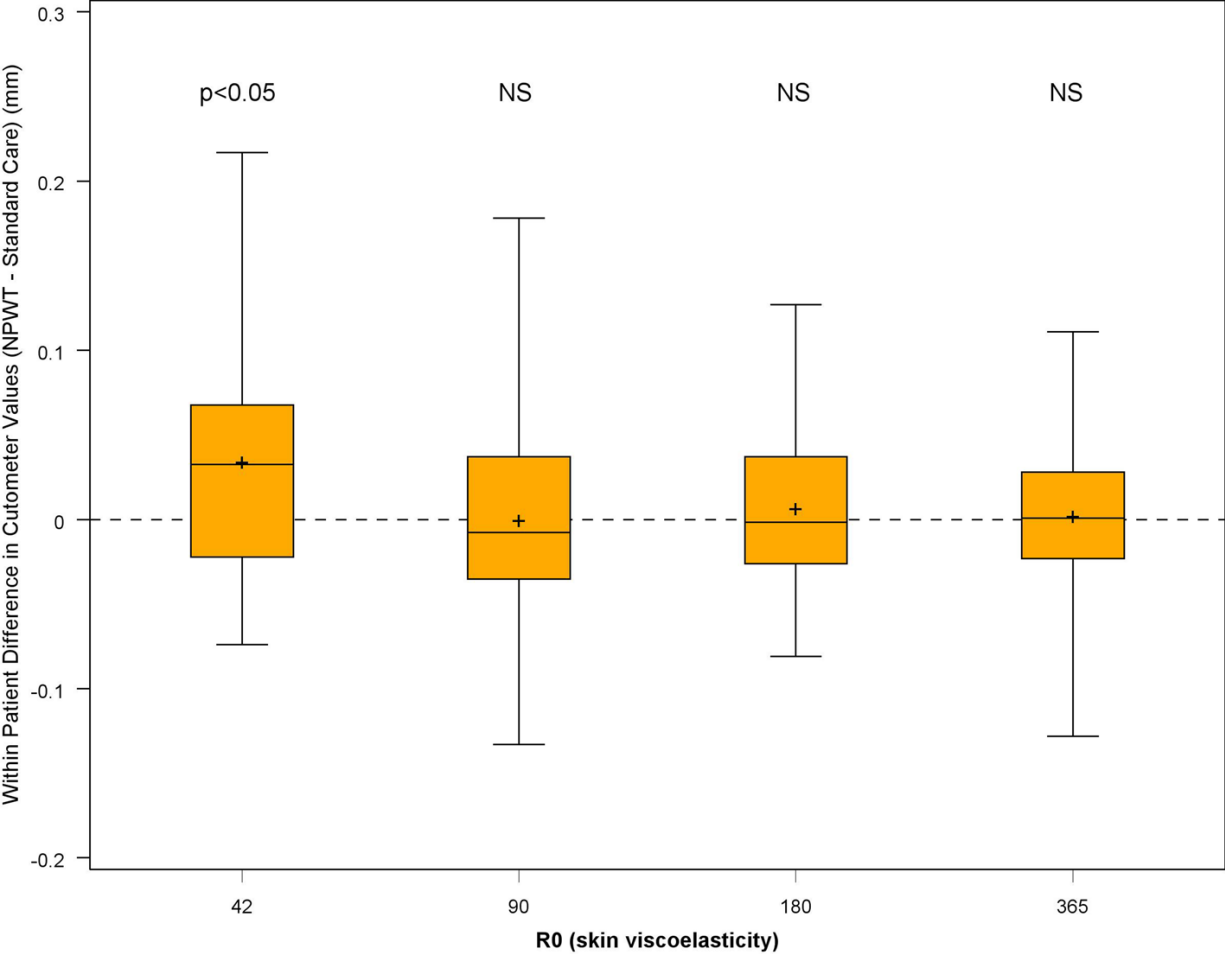
*N* = 32 skin viscoelasticity scored at 42, 90, 180 and 365 days

**Fig. 6:**
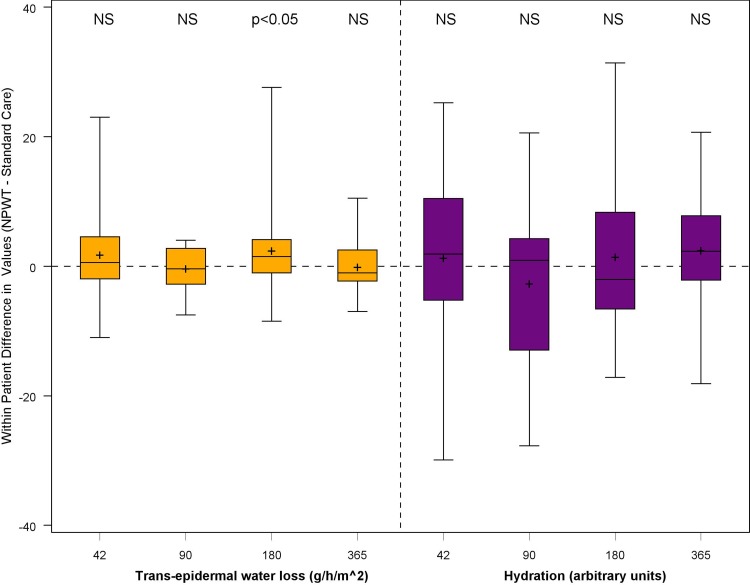
*N* = 32 trans-epidermal water loss and skin hydration

**Fig. 7:**
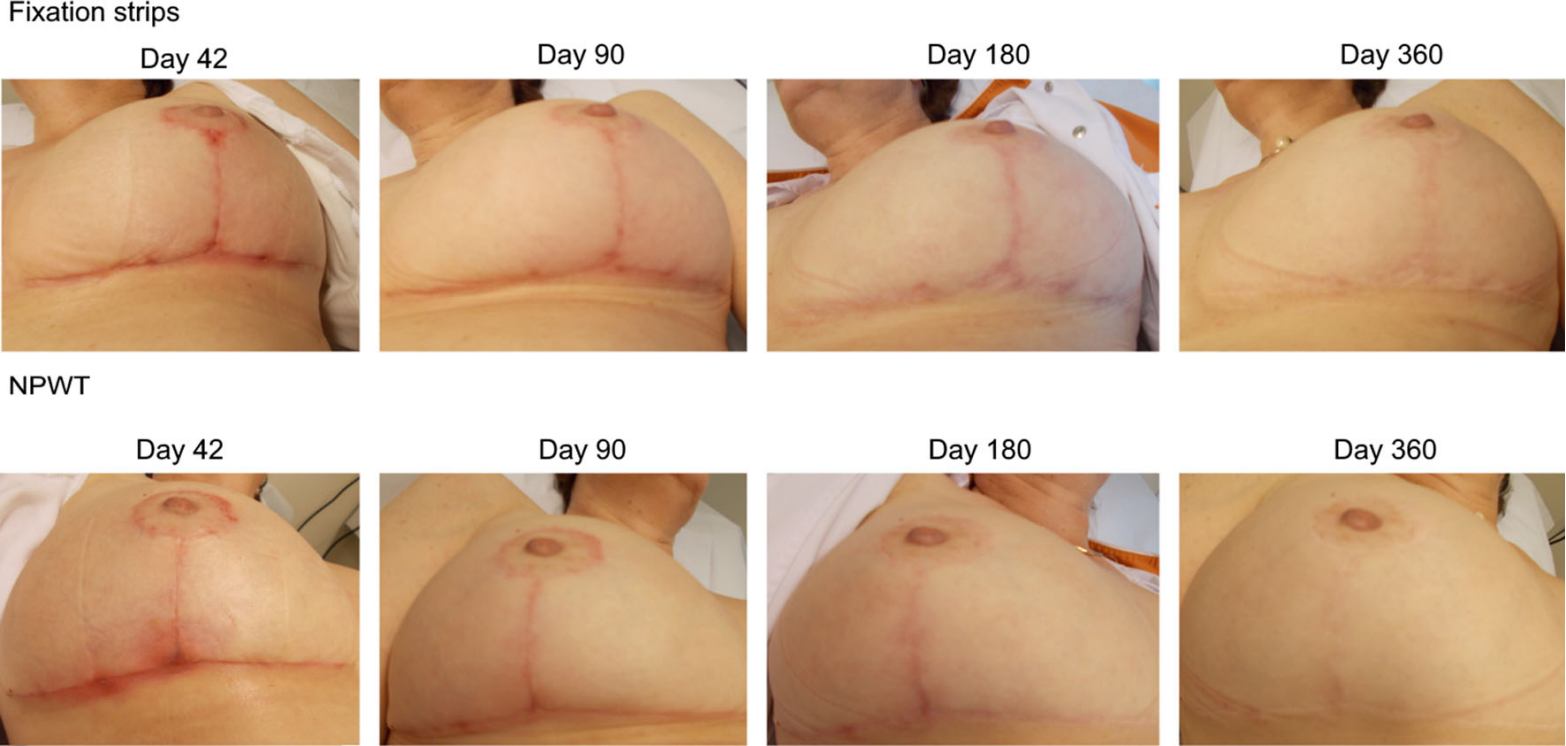
Case 42, 90, 180 and 365 days

The scar quality and aesthetic appearance as assessed using POSAS (Fig. 3) and VAS (Fig. 4) at 42, 90 and 180 days revealed a significantly (*p* < 0.05) better quality of scarring in the NPWT treatment breasts compared to standard care, using fixation strips. Skin viscoelasticity (Fig. 5), trans-epidermal water loss and hydration measurements (Fig. 6) at follow-up visits day 42, 90, 180 and 365 showed no consistent significant improvement. The results are illustrated in a typical case (Fig. 7). NPWT was easy to use. Patients reported the tested NPWT system to be comfortable to wear. NPWT removal did not cause much pain or skin irritation. The overall acceptance of the NPWT system both by physicians and by patients scored high.

## Discussion

NPWT refers to the controlled application of sub-atmospheric pressure to promote wound healing in a wide array of acute and complex wounds [7–9]. Since its introduction into clinical practice in the late 1990s, it has been advocated for use on various types of ulcers [7–9, 23], open fractures and other posttraumatic wounds [24, 25], acute burns [26], split thickness skin grafts [27], open abdominal wounds [28] and sternal wounds [29]. Over the years, the clinical success of NPWT encouraged some specialists to apply the technique to closed surgical incisions. It serves as a preventative measure after high-risk procedures and patients at risk for postsurgical complications [10, 11, 29]. Stannard et al. [12, 13] published two studies on different high-risk skeletal traumas in which they concluded that there were significantly less infections and cases of dehiscence in the NPWT group compared to standard care. A retrospective review of 57 patients who received NPWT postoperatively after a sternotomy by Atkins et al. [30] showed no superficial wound infections while they were in a high-risk group. Favorable outcomes were also presented by Reddix et al. [31, 32] in a series of 19 morbidly obese patients who received surgery for acetabular fractures and later in a large group of patients who underwent the same procedure. A Cochrane review specifically focused on studies involving the use of NPWT on closed incisions and skin grafts found no significant evidence for its effectiveness. The authors stated: “given the cost and widespread use of NPWT, there is an urgent need for suitably powered, high-quality trials to evaluate the effects of the newer NPWT products that are designed for use on clean, closed surgical incisions” [33].

For this purpose, our study selected bilateral reduction mammoplasty patients who are at risk for postoperative wound complications [1–6]. The study included thirty-two patients, who served as their own control. The patients were suitable candidates for incisional NPWT treatment and standard care using fixation strips. In line with a wider RCT published recently [34], this study showed significantly fewer wound healing complications had occurred in the NPWT treated sites, compared to standard care with fixation strips at 7 and at 21 days post-surgery assessment. Treatment with NPWT resulted in significantly lower incidence of dehiscence compared to standard care. Scar quality as scored by VAS and POSAS was shown to be significantly better for the NPWT treated sites than for fixation strips treated sites. However, no consistent significant improvement in scar viscoelasticity was demonstrated.

Cost of wounds is measured in pain, distress, embarrassment, anxiety and prolonged hospital stay. Further costs are related to wound infection, increased levels of exudate, pain and odor and prolonged inflammation, frequently resulting in further surgical interventions and poor scarring. Surgical infection affects 30–40 patients per 1000 operations with a mean additional length of stay of 11 days [35]. It was beyond the scope of the paper to report on cost implications of the NPWT treatment that was used. Cost efficacy, specifically on prevention of wound dehiscence, will be covered in a further publication.

### Limitations

Surgical technique is a recognized factor in post-surgery wound healing [2–4]. POSAS and VAS scores showed a better quality of scarring in the NPWT-treated sites compared to fixation strips, indicating wound healing was mainly uneventful. Given the nature of the treatments, it was not possible to conduct a double-blind trial. Ideally, a control group with the same non-activated device would have been better, but this would still have been noted by the patient and physician. Therefore, investigator and patient bias scoring POSAS and VAS cannot be ruled out. Our intention was to at least blind the investigator, which was unsuccessful due to practical reasons, e.g., the assessor was not available. Instead, the investigator did not know the randomization schedule and the patients were asked not to reveal it. No consistent significant improvement in scar viscoelasticity was demonstrated. This could be due to measurements being performed with different probes and other external factors influencing results. Even after standardizing measurement locations, it proved difficult to exactly measure the same section of scar/skin during follow-up. Moreover, some patients had such a fine scar that the probes were overlapping onto normal skin. Although we tried to keep a steady room temperature and air humidity, thereby minimizing environmental factors, this was practically impossible at the outpatient clinic. During the follow-up period, seasons changed making the comparison of TEWL in serial follow-ups difficult. Some patients were stressed because they were late or had put on body lotion when they were not supposed to.

## Conclusions

Our study showed less wound healing complications and a statistically significant improvement in the aesthetic appearance and quality of scarring for the NPWT-treated sites versus those breasts that received standard care with fixation strips. The results indicate NPWT to be an attractive option for closed surgical incision treatment. Being able to minimize scarring as well as postsurgical outcomes has great clinical significance to the patient. Although the clinical significance of reduced scarring may be not of utmost importance for the surgeon, it is imperative for the patient’s emotional well-being and quality of life.

## References

[1] Shermak MA, Chang D, Buretta K, Mithani S, Mallalieu J, Manahan M (2011). Increasing age impairs outcomes in breast reduction surgery. Plast Reconstr Surg.

[2] Eggert E, Schuss R, Edsander-Nord A (2009). Clinical outcome, quality of life, patients’ satisfaction, and aesthetic results, after reduction mammaplasty. Scand J Plast Reconstr Surg Hand Surg.

[3] Cunningham BL, Gear AJ, Kerrigan CL, Collins ED (2005). Analysis of breast reduction complications derived from the BRAVO study. Plast Reconstr Surg.

[4] Henry SL, Crawford JL, Puckett CL (2009). Risk factors and complications in reduction mammaplasty: novel associations and preoperative assessment. Plast Reconstr Surg.

[5] Makki AS, Ghanem AA (1998). Long-term results and patient satisfaction with reduction mammaplasty. Ann Plast Surg.

[6] Meshulam-Derazon S, Barnea Y, Zaretski A, Leshem D, Miller U, Meilik B (2009). Large-volume breast reduction: long-term results. Scand J Plast Reconstr Surg Hand Surg.

[7] Argenta LC, Morykwas MJ (1997). Vacuum-assisted closure: a new method for wound control and treatment: clinical experience. Ann Plast Surg.

[8] Armstrong DG, Lavery LA, Diabetic Foot Study Consortium (2005). Negative pressure wound therapy after partial diabetic foot amputation: a multicentre, randomised controlled trial. Lancet.

[9] Mandal A (2007). Role of topical negative pressure in pressure ulcer management. J Wound Care.

[10] Morykwas MJ, Simpson J, Punger K, Argenta A, Kremers L, Argenta J (2006). Vacuum-assisted closure: state of basic research and physiologic foundation. Plast Reconstr Surg.

[11] Wilkes RP, Kilpad DV, Zhao Y, Kazala R, McNulty A (2012). Closed incision management with negative pressure wound therapy (CIM): biomechanics. Surg Innov.

[12] Stannard JP, Robinson JT, Anderson ER, McGwin G, Volgas DA, Alonso JE (2006). Negative pressure wound therapy to treat hematomas and surgical incisions following highenergy trauma. J Trauma.

[13] Stannard JP, Volgas DA, McGwin G (2012). Incisional negative pressure wound therapy after high-risk lower extremity fractures. J Orthop Trauma.

[14] Hudson DA, Adams KG, Huyssteen AV, Martin R, Huddleston EM (2013). Simplified negative pressure wound therapy: clinical evaluation of an ultraportable, no-canister system. Int Wound J.

[15] Draaijers LJ (2004). The patient and observer scar assessment scale: a reliable and feasible tool for scar evaluation. Plast Reconstr Surg.

[16] van de Kar AL (2005). Reliable and feasible evaluation of linear scars by the patient and observer scar assessment scale. Plast Reconstr Surg.

[17] Duncan JA (2006). Visual analogue scale scoring and ranking: a suitable and sensitive method for assessing scar quality?. Plast Reconstr Surg.

[18] Durani P, McGrouther DA, Ferguson MW (2009). Current scales for assessing human scarring: a review. J Plast Reconstr Aesthet Surg.

[19] Micomonaco DC (2009). Development of a new visual analogue scale for the assessment of area scars. J Otolaryngol Head Neck Surg.

[20] Enomoto DN (1996). Quantification of cutaneous sclerosis with a skin elasticity meter in patients with generalized scleroderma. J Am Acad Dermatol.

[21] Fong SS, Hung LK, Cheng JC (1997). The cutometer and ultrasonography in the assessment of postburn hypertrophic scar—a preliminary study. Burns.

[22] Draaijers LJ (2004). Skin elasticity meter or subjective evaluation in scars: a reliability assessment. Burns.

[23] Suissa D, Danino A, Nikolis A (2011). Negative-pressure therapy versus standard wound care: a meta-analysis of randomized trials. Plast Reconstr Surg.

[24] Stannard JP, Volgas DA, Stewart R, McGwin G, Alonso JE (2009). Negative pressure wound therapy after severe open fractures: a prospective randomized study. J Orthop Trauma.

[25] Kanakaris NK, Thanasas C, Keramaris N, Kontakis G, Granick MS, Giannoudis PV (2007). The efficacy of negative pressure wound therapy in the management of lower extremity trauma: review of clinical evidence. Injury.

[26] Molnar JA, Simpson JL, Voignier DM, Morykwas MJ, Argenta LC (2005). Management of an acute thermal injury with subatmospheric pressure. J Burns Wounds.

[27] Llanos S, Danilla S, Barraza C, Armijo E, Piñeros JL, Quintas M, Searle S, Calderon W (2006). Effectiveness of negative pressure closure in the integration of split thickness skin grafts: a randomized, double masked, controlled trial. Ann Surg.

[28] Stevens P (2009). Vacuum-assisted closure of laparotomy wounds: a critical review of the literature. Int Wound J.

[29] Sjogren J, Gustafsson R, Nilsson J, Lindstedt S, Nozohoor S, Ingemansson R (2011). Negative-pressure wound therapy following cardiac surgery: bleeding complications and 30-days mortality in 176 patients with deep sternal wound infection. Interact CardioVasc Thorac Surg.

[30] Atkins BZ, Wooten MK, Kistler J, Hurley K, Hughes GC, Wolfe WG (2009). Does negative pressure wound therapy have a role in preventing poststernotomy wound complications?. Surg Innov.

[31] Reddix RN, Tyler HK, Kulp B, Webb LX (2009). Incisional vacuum-assisted wound closure in morbidly obese patients undergoing acetabular fracture surgery. Am J Orthop.

[32] Reddix RN, Leng XI, Woodall J, Jackson B, Dedmond B, Webb LX (2010). The effect of incisional negative pressure therapy on wound complications after acetabular fracture surgery. J Surg Orthop Adv.

[33] Webster J, Scuffham P, Sherriff KL, Stankiewicz M, Chaboyer WP (2012) Negative pressure wound therapy for skin grafts and surgical wounds healing by primary intention. Cochrane Database Syst Rev. 10.1002/14651858.CD009261.pub2 10.1002/14651858.CD009261.pub2 22513974

[34] Galiano RD, Hudson D, Shin J, van der Hulst R, Tanaydin V, Djohan R, Duteille F, Cockwill J, Megginson S, Huddleston E (2018) Incisional negative pressure wound therapy for prevention of wound healing complications following reduction mammaplasty. Plast Reconstr Surg Global Open. 10.1097/GOX.0000000000001560 10.1097/GOX.0000000000001560 PMC5811280 29464150

[35] Posnett J, Gottrup F, Lundgren H, Saal G (2009). The resource impact of wounds on healthcare providers in Europe. J Wound Care.

